# Borna Disease Virus Infection Perturbs Energy Metabolites and Amino Acids in Cultured Human Oligodendroglia Cells

**DOI:** 10.1371/journal.pone.0044665

**Published:** 2012-09-07

**Authors:** Rongzhong Huang, Hongchang Gao, Liang Zhang, Jianmin Jia, Xia Liu, Peng Zheng, Lihua Ma, Wenjuan Li, Jing Deng, Xiao Wang, Liu Yang, Mingju Wang, Peng Xie

**Affiliations:** 1 Department of Neurology, The First Affiliated Hospital, Chongqing Medical University, Chongqing, China; 2 Department of Pharmacy, Wenzhou Medical College, Wenzhou, Zhejiang, China; 3 Chongqing Key Laboratory of Neurobiology, Chongqing, China; 4 Institute of Neuroscience, Chongqing Medical University, Chongqing, China; The Scripps Research Institute, United States of America

## Abstract

**Background:**

Borna disease virus is a neurotropic, non-cytolytic virus that has been widely employed in neuroscientific research. Previous studies have revealed that metabolic perturbations are associated with Borna disease viral infection. However, the pathophysiological mechanism underlying its mode of action remains unclear.

**Methodology:**

Human oligodendroglia cells infected with the human strain Borna disease virus Hu-H1 and non-infected matched control cells were cultured *in vitro*. At day 14 post-infection, a proton nuclear magnetic resonance-based metabonomic approach was used to differentiate the metabonomic profiles of 28 independent intracellular samples from Borna disease virus-infected cells (n = 14) and matched control cells (n = 14). Partial least squares discriminant analysis was performed to demonstrate that the whole metabonomic patterns enabled discrimination between the two groups, and further statistical testing was applied to determine which individual metabolites displayed significant differences between the two groups.

**Findings:**

Metabonomic profiling revealed perturbations in 23 metabolites, 19 of which were deemed individually significant: nine energy metabolites (α-glucose, acetate, choline, creatine, formate, myo-inositol, nicotinamide adenine dinucleotide, pyruvate, succinate) and ten amino acids (aspartate, glutamate, glutamine, glycine, histidine, isoleucine, phenylalanine, threonine, tyrosine, valine). Partial least squares discriminant analysis demonstrated that the whole metabolic patterns enabled statistical discrimination between the two groups.

**Conclusion:**

Borna disease viral infection perturbs the metabonomic profiles of several metabolites in human oligodendroglia cells cultured *in vitro*. The findings suggest that Borna disease virus manipulates the host cell’s metabolic network to support viral replication and proliferation.

## Introduction

Borna disease virus (BDV) [1], a neurotropic, non-cytolytic, non-segmented, negative-stranded non-retroviral RNA virus, has been widely employed in neuroscientific research on account of these unique attributes [2]. A previous study by this group has demonstrated that there may be an association between BDV infection and human viral encephalitis [3]. However, the mechanism underlying BDV pathogenesis itself, most notably the influence of BDV infection on host cell metabolism, is not well understood. Previous studies in animal models have demonstrated that BDV infection affects the host’s neuroendocrine systems [4]. Notably, BDV infection perturbs levels of the hormones cholecystokinin and somatostatin *in vivo*. The virus also manipulates cholinergic, GABAergic, and monoaminergic neurotransmitter pathways, as significant alterations in choline acetyltransferase (ChAT), acetylcholinesterase (AchE), glutamic acid decarboxylase (GAD), norepinephrine, and serotonin levels have been reported [5]. Of particular interest are the congruent hippocampal distribution patterns of BDV proteins and the kainite 1 (KA-1) glutamate receptor found in persistently infected rats with learning deficiencies [6], [7]. However, the intracellular metabolic alterations associated with BDV infection have not yet been systematically profiled in human cells.

Metabonomics enables the simultaneous quantitative measurement of numerous low molecular weight molecules within a particular sample [8], [9]. Metabolic profiling techniques, such as gas/liquid chromatography-mass spectrometry (GC-MS) and nuclear magnetic resonance (NMR) coupled with multivariate statistical modeling, have been used to analyze the changes in whole metabolic patterns in response to non-physiologic challenges such as viral infection [10], [11]. Due to its strong resolution and sensitivity, a NMR-based metabonomic approach instituting high-throughput molecular screening has already demonstrated promising results in metabolite identification and quantification in mammalian cell lines [12].

Cell culture has been successfully applied in metabonomics to enable the investigation of metabolic changes on a cellular and subcellular level. Oligodendrocytes, a widely-used neuronal cell line employed here as an Mycoplasma-free oligodendroglia cell line originally introduced to study BDV and isolate the virus from human white blood cells [13], are a major cellular component of the CNS white matter that play a pivotal role in maintaining neurological function. Oligodendrocytes have been shown to support both natural and artificial infection with several neurotropic NNS RNA viruses (e.g., canine distemper virus [14], measles virus [15], and BDV [16], [17]), revealing that oligodendrocytes are a target of these viruses and may be involved in their pathogenic mechanism.

The current study describes the application of a ^1^H NMR-based metabonomic method in differentiating the metabolic profiles of human oligodendroglia (OL) cells infected with BDV Hu-H1 and matched control cells *in vitro*. The primary objective of this study is to assess whether metabonomic analysis can statistically distinguish the metabolic expression between infected and uninfected cells in order to elucidate BDV’s effect on intracellular metabolic pathways. A secondary objective is to provide information for future studies on the pathogenic mechanisms of BDV, considering the reported integration of BDV genome fragments into mammalian genomes [18], [19].

## Results

### Immunoﬂuorescence Assay

**Figure 1:**
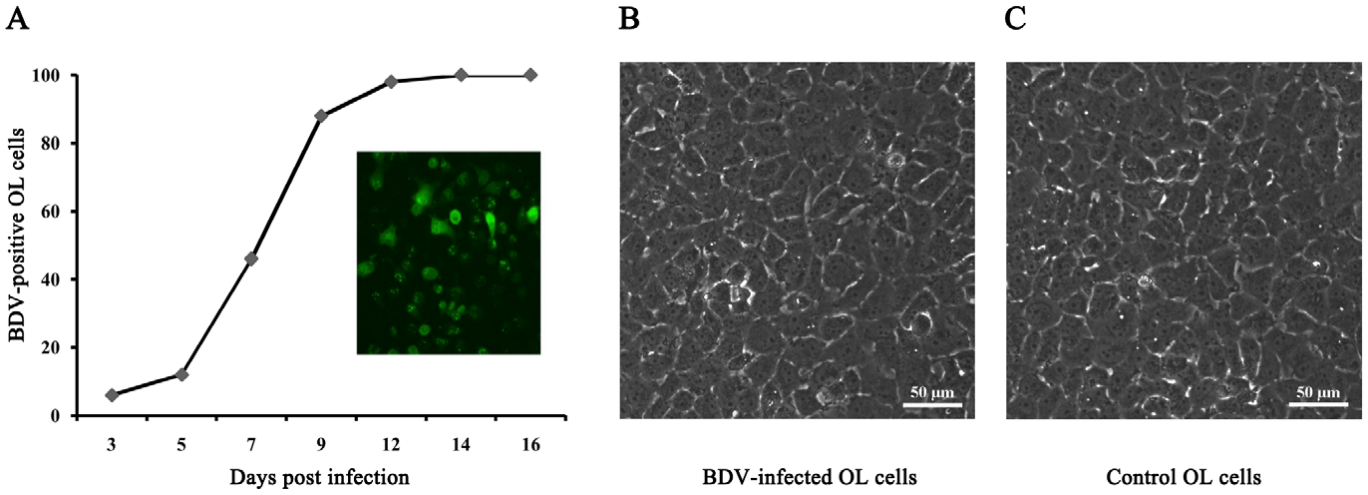
OL cells were labeled for BDV-specific nucleoprotein p40 and analyzed by immunofluorescence. The inset photo shows OL cells stained for the BDV antigen at 14 days post-infection (A). Representative phase-contrast micrographs of control OL cells (C) and BDV-infected OL cells (B). There were no visible changes in cell structure. Scale bar: 50 µm.

The immunofluorescence assay was applied to stain BDV-specific nucleoprotein p40 at days 3, 5, 7, 9, 12 and 14 post-infection (Fig. 1A). Rapid spread of the virus was observed in tissue culture; 100% of the cells were infected at day 14. The percentage of labeled OL cells was determined through observation of a minimum of 300 randomly selected cells across three independent experiments. BDV infection had no discernible cytopathic effect in these human OL in cell monolayers, as evidenced by phase-contrast microscopy (Fig. 1B and 1C).

### Metabonomic Profiling

**Figure 2:**
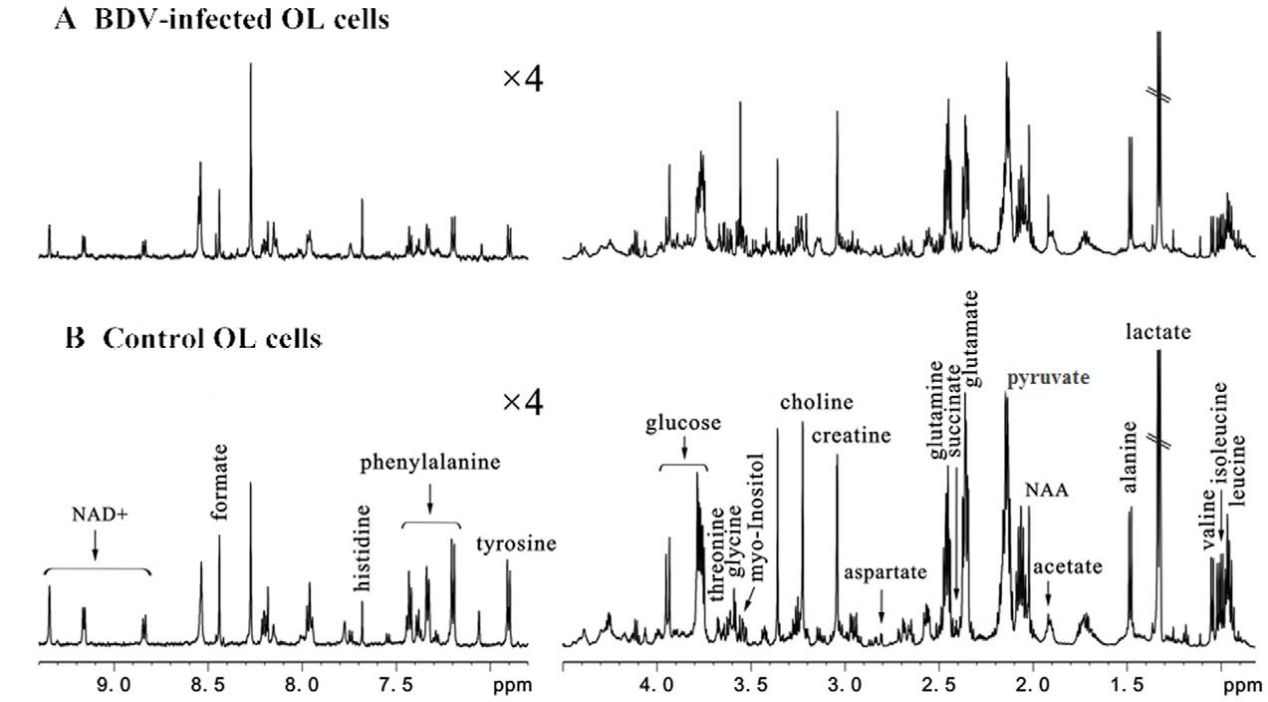
Representative 600 MHz 1D 1H NMR spectra indicating the key metabolites extracted from intracellular samples of BDV-infected OL cells (A) and control OL cells (B).

**Figure 3:**
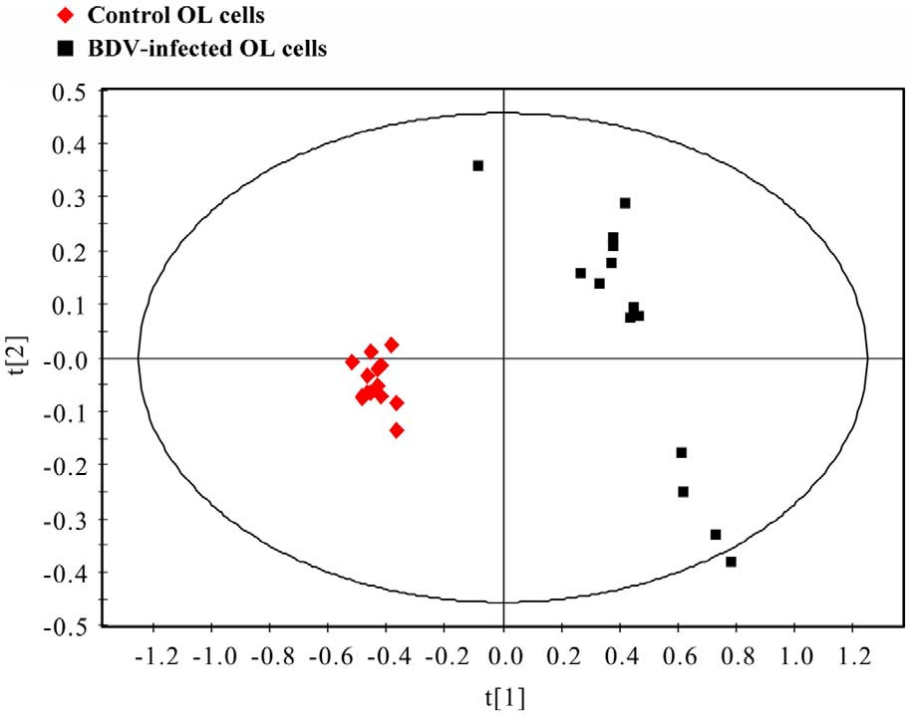
PLS-DA score plots displaying discrimination between BDV-infected OL cells (▪) and control OL cells (♦).

600 MHz 1D ^1^H NMR spectra were obtained from both BDV-infected and control OL cells (Fig. 2). The resonance assignments (Fig. 2) were selected based on current literature and verified by 2D ^1^H–^1^H COSY and TOCSY spectra. Metabonomic analysis was then applied to statistically differentiate BDV-infected and control OL cells on a whole metabolic pattern basis. First, partial least squares discriminant analysis (PLS-DA) was applied to discriminate between the two groups (n = 28 independent samples; n = 14 per group). The PLS-DA score plots revealed that the BDV-infected OL cells were significantly distinguishable from control OL cells (R^2^X = 0.821, R^2^Y = 0.842, Q^2^ = 0.556; Fig. 3). All descriptive parameters were significantly elevated (R^2^X, R^2^Y, Q^2^>0.5), demonstrating that the PLS-DA model was robust (Fig. 3).

**Figure 4:**
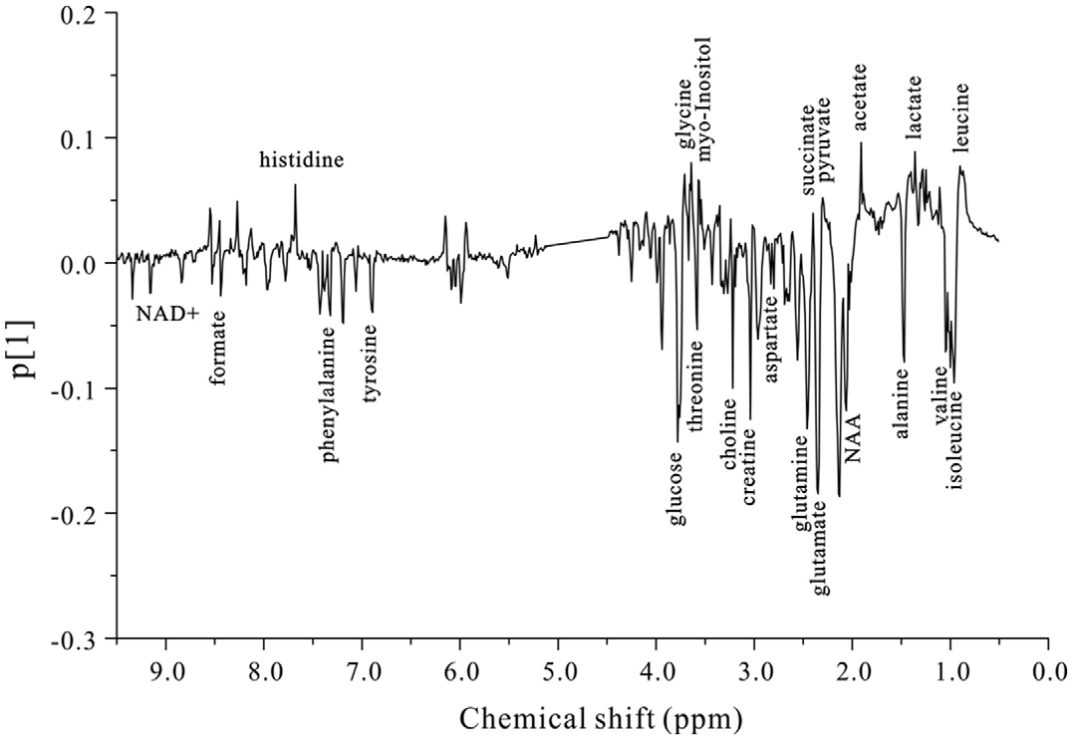
PLS-DA loading plot demonstrating discrimination of 23 key metabolites between BDV-infected OL cells and control OL cells.

PLS-DA loading plot analysis (Fig.4) revealed that 23 key metabolites were primarily responsible for the score plot discrimination between the two groups. As compared to control OL cells, BDV-infected OL cells were characterized by perturbed levels in 11 energy metabolism-related molecules (α-glucose, acetate, choline, creatine, formate, lactate, myo-inositol, N-acetylaspartate (NAA), nicotinamide adenine dinucleotide (NAD+), pyruvate, and succinate) and 12 amino acids (alanine, aspartate, glutamate, glutamine, glycine, histidine, isoleucine, leucine, phenylalanine, threonine, tyrosine, and valine). The spectral resonances assigned to these key metabolites are noted (Fig. 2B). A two-tailed Student’s *t*-test and non-parametric Mann-Whitney U-test test (*p*-value) were then applied to determine significant differences on a metabolite-by-metabolite basis; levels of 19 of the mentioned 23 metabolites were found to be significantly different between infected and uninfected OL cells after controlling for the false discovery rate (Table 1).

**Table 1:** 23 Key Metabolites Differentiating BDV-infected and Control OL Cells Derived from the PLS-DA Model.

Metabolite	Chemical Shift (ppm)	Relative Peak Intensity (x 10^∧^-4)		Fold Δ(↑↓)	P-value	Q-value^e^
		Control OL cells^b^	BDV-infected OL cells^b^			
Valine	1.061-1.032	43±3.5	79±4.6	1.84↑	7.98E-19^d^	1.84E-17
Tyrosine	6.913-6.885	11±4.3	28±1.7	2.55↑	3.48E-13^d^	4.00E-12
Phenylalanine	7.351-7.312	11±2.7	20±0.9	1.82↑	1.10E-11^d^	8.42E-11
Pyruvate	2.411-2.397	13±2.0	7±0.5	0.54↓	2.33E-10^d^	1.07E-09
Threonine	3.596-3.578	16±4.1	28±2.1	1.75↑	2.24E-10^d^	1.29E-09
Creatine	3.050-3.032	29±9.1	54±3.7	1.86↑	4.46E-10^d^	1.71E-09
Acetate	1.926-1.914	27±9.2	13±1.3	0.48↓	3.08E-06^d^	1.01E-05
Glutamate	2.392-2.333	56±22.2	177±11.9	3.16↑	6.70E-06^c^	1.71E-05
Isoleucine	1.027-1.005	41±4.1	73±4.5	1.78↑	6.70E-06^c^	1.93E-05
NAD+^a^	9.171-9.146	7±3.2	13±1.5	1.86↑	1.03E-05^c^	2.15E-05
Choline	3.235-3.218	19±5.2	38±8.9	2.00↑	1.03E-05^c^	2.37E-05
Glycine	3.563-3.550	21±9.1	12±1.3	0.57↓	1.57E-05^c^	3.00E-05
Myo-inositol	3.550-3.517	86±23.2	56±7.9	0.65↓	1.14E-04^c^	1.87E-04
Glutamine	2.479-2.422	70±24.3	123±4.0	1.76↑	1.14E-04^c^	2.01E-04
Histidine	7.688-7.676	57±45.6	13±2.8	0.23↓	1.98E-04^c^	3.03E-04
Formate	8.452-8.430	8±3.9	13±1.4	1.63↑	5.69E-04^c^	8.18E-04
α-Glucose	5.247-5.223	15±16.5	7±4.1	0.47↓	7.70E-03^c^	9.84E-03
Aspartate	2.818-2.794	18±9.9	20±2.3	1.11↑	7.70E-03^c^	1.04E-02
Succinate	2.417-2.411	5±0.5	6±0.4	1.20↑	3.87E-02^c^	4.68E-02
Lactate	1.350-1.315	187±45.0	166±10.9	0.89↓	5.36E-02^c^	6.17E-02
Leucine	0.950-0.924	74±13.1	69±4.1	0.93↓	3.12E-01^c^	3.26E-01
Alanine	1.496-1.461	35±8.7	54±4.4	1.54↑	3.31E-01^c^	3.31E-01
NAA^a^	2.025-2.014	22±12.8	24±1.8	1.09↑	3.12E-01^c^	3.41E-01

*Note: The 19 individually significant differential metabolites (p,q<0.05) are bolded for emphasis.*

a NAA: N-acetylaspartate, NAD^+^: Nicotinamide adenine dinucleotide.

b Values expressed as means ± SD’s.

c Non-parametric Mann-Whitney U test.

d Student *t*-test.

e Q-values were calculated by the Benjamini-Hochberg method.

## Discussion

BDV is an evolutionary old neurotropic, non-cytolytic RNA virus infecting humans, non-human primates, and other mammalian species. The virus persistently infects brain and blood cells and has integrated its genome fragments into host genomes [1], [18], [19], [20]. BDV’s controversial implication in mood disorders (e.g., bipolar disorder, major depressive disorder) [13] has stimulated worldwide research, but whether and to what extent BDV infection influences the development of any human neuropsychiatric illness remains non-conclusive partly on account of the different diagnostic approaches involved (ELISA, Western blotting) [13], [21]. However, given its unique neurotropic and non-lytic properties, BDV is still regarded as an attractive model for investigating brain cell function [22]. Previous studies have demonstrated that BDV infection is associated with perturbations in several metabolic pathways [4], [5]. However, the molecular basis of BDV pathogenesis remains largely unknown and requires further elucidation. The current study is the first to use ^1^H NMR spectroscopy in analyzing BDV-infected intracellular samples.

In this study, a^ 1^H NMR-based metabonomic method was employed to characterize the metabolic changes inside BDV-infected OL cells. A panel of metabolites differentiating BDV-infected and control OL cells were identified at day 14 post-infection; these metabolic “signatures” represent the total sum of intracellular biochemical reactions. Therefore, statistically significant differences in these signatures provide insight into the metabolic pathways affected by BDV; these findings may contribute to a better understanding of BDV’s pathogenic mode of action. One striking observation concerning the PLS-DA score plot (Fig. 3) is the clearly visible separation between BDV-infected OL cells and control OL cells. The dispersion in metabolic profiles among BDV-infected cells is considerably greater than those among healthy control cells in the PLS-DA score plot. This finding may reflect a metabolic “instability” subsequent to BDV infection. As discussed below, significant changes in metabolite levels were observed in i) energy metabolites and ii) amino acids.

### Energy Metabolites

**Figure 5:**
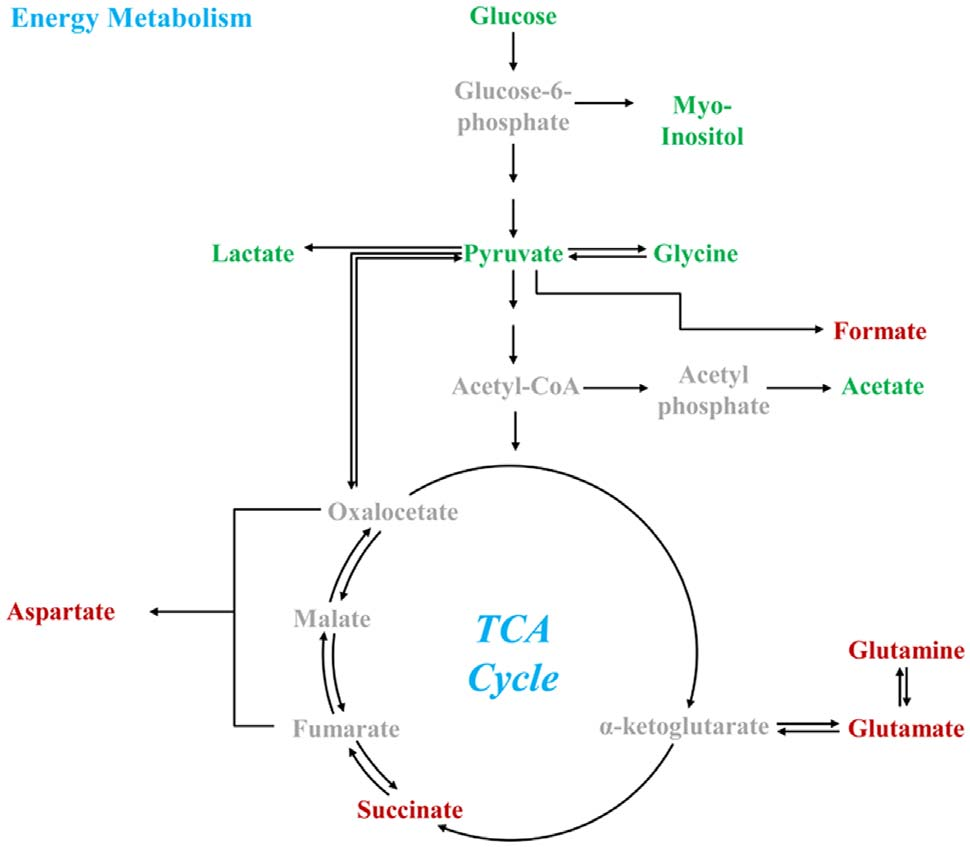
Key metabolites of energy production pathway. Red indicates upregulation; green indicates downregulation.

In order to drive adenosine triphosphate (ATP) production, α-glucose is glycolytically converted to pyruvate, which is then aerobically catabolized through the TCA cycle/respiratory chain or anaerobically fermented into lactate [23], [24], [25], [26], [27] (Fig. 5). Previous studies have demonstrated that several viral infectious agents disturb both glycolysis and the TCA cycle [11]. In the present study, glucose, myo-inositol, pyruvate, glycine, and acetate levels were all significantly downregulated in BDV-infected OL cells as compared to controls. Moreover, formate, succinate, aspartate, glutamate, and glutamine levels were significantly upregulated in BDV-infected cells as compared to controls [28], [29]. As formate is a by-product of formate C-acetyltransferase’s production of acetyl-CoA from pyruvate, it is reasonable to deduce that acetyl-CoA levels (although not directly measurable by the NMR method employed here) were also elevated. Therefore, the lower levels of glycolytic intermediates and their respective by-products in conjunction with the higher levels of acetyl-CoA, TCA intermediates, and their respective by-products indicate a downstream equilibrium shift away from glycolysis and increased carbon flux through the TCA cycle in BDV-infected cells. This shunting toward the TCA cycle is consistent with the decreases in acetate and lactate levels; although the change in lactate was not statistically significant, it is consistent with a shunting away from the anaerobic pathway toward the aerobic pathway in BDV-infected cells [30].

According to Ritter, the replication of any virus requires ATP for the synthesis of macromolecules like proteins or, depending on the virus, DNA or RNA. Additionally, in the case of a lipid-enveloped virus, viral budding requires de novo synthesis of membrane lipids [31]. Therefore, BDV may be manipulating these pathways to i) produce ATP more efficiently, as the aerobic pathway is approximately 15x more efficient at ATP production than the anaerobic pathway, and/or ii) promote de novo synthesis of membrane lipids, as BDV’ particles are enlosed by a 130 nm-diameter lipid envelope that enables budding off the cell surface [32]. As BDV grows to relatively low titres in persistently infected cells with an infectious particle-to-cell ratio of a mere 0.01 to 0.05, ATP requirements for viral particle replication are not particularly demanding relative to most other viruses [33]. However, intra-nuclear replication, a unique feature of BDV within the order Mononegavirales [2], is associated with frequent ATP-consuming nuclear protein import/export mechanisms. Thus, frequent protein trafficking between the nucleus and cytoplasm, together with the expression of BDV’s N- and P-proteins, may account for the shift to the more energy-efficient aerobic pathway observed in BDV-infected cells.

In addition, this set of metabolic perturbations may have also evolved to promote de novo synthesis of membrane lipids, as has been previously demonstrated for the lipid-enveloped human cytomegalovirus (CMV), [34] through increases in acetyl-CoA and decreases in myo-inositol – a byproduct of membrane-bound phospholipid metabolism – in BDV-infected cells In a previous study, acetyl-CoA has been reported as a carbon contributor to *de novo* lipid biosynthesis in CMV-infected cells [11]. Moreover, the decreased level of free acetate found in BDV-infected cells accounts for decreased lipolysis/increased liposynthesis, as free acetate is produced *in vivo* via lipid oxidation. This finding correlates well with the elevated lipid levels previously observed in BDV-infected cells [35]. The downregulation of acetate and myo-inositol, combined with the decreased levels of free acetate, likely indicate a more lipid-rich microenvironment conducive to improved viral envelope development and budding. In addition, BDV also depends on glycoprotein-mediated attachment to BDV-susceptible cells; specifically, this glycoprotein localizes to lipid and aids BDV’s entry into cells [32], [35]. Thus, BDV’s lipid envelope is important for viral budding as well as glycoprotein-mediated cellular entry – both important elements in viral proliferation. As little research has been done on this specific issue, further investigation is necessary to measure and validate the precise metabolic changes affecting *de novo* lipid biosynthesis and their relationship to viral proliferation in BDV and other lipid-enveloped viruses.

### Amino Acids

Levels of several amino acids were perturbed in BDV-infected cells as compared to controls. Most notably, amino acid metabolic profiling detected significant changes in three non-aromatic amino acids that function as CNS neurotransmitters (aspartate, glutamate, and glycine) and two aromatic amino acids (phenylalanine and tyrosine) that are upstream precursors to two other CNS neurotransmitters, dihydroxyphenylalanine and dopamine [36]. Without regard to the etiopathology of any neurological disease state, this altered amino acid profile is noteworthy in light of previous studies linking other neurotropic viral infections with CNS neurotransmitter dysregulation [37].

In the mammalian CNS, glutamate and aspartate act as the primary excitatory neurotransmitters, while glycine functions as a primary inhibitory neurotransmitter [38], [39]. Previous studies in a rat model showed that perturbations in these three neurotransmitters led to over-activation of the N-methyl-D-aspartate (NMDA) receptor, inducing excitotoxicity and neurodegeneration in the hippocampal CA1 region [40], [41]. The hippocampal distribution pattern of BDV’s proteins and RNA is restricted to the CA3 region and the dentate gyrus and is absent in the CA1 region. These findings point to the kainite 1 (KA-1) glutamate receptor that has been shown to produce an immunohistologic staining pattern congruent with those of BDV’s N- and P-proteins. This staining pattern, in contrast to the NMDA receptor, are also notably absent in the CA1 region [6]. The current findings are supported by a previous *in vivo* neonatal rat brain study that reported higher glutamate levels in BDV-infected subjects, but contrasted on aspartate levels (Fig. 5) [42]. As the current study was performed *in vitro*, this difference may be explained by a different metabolic processing of aspartate *in vivo*.

Both phenylalanine and tyrosine levels were found to be elevated in BDV-infected cells as compared to controls. A previous study, employing a high performance liquid chromatography (HPLC)-based metabonomic approach, found that the CNS neurotransmitters dihydroxyphenylalanine and dopamine were significantly upregulated in various brain regions of a BDV-infected rat model [5]. Although not measured in this *in vitro* study, the upregulation in dihydroxyphenylalanine and dopamine found in the previous *in vivo* work may be related to the intracellular upregulation of phenylalanine and tyrosine found here. Further investigation is required to shed light on the relationships between BDV infection, the foregoing neurotransmitters, their receptors, and their respective regulatory pathways, particularly with respect to these pathways’ possible interactions with BDV proteins.

### Conclusion

In the current study, a ^1^H NMR-based metabonomic method was applied to differentiate the metabolic profiles of human strain BDV Hu-H1-infected human oligodendroglia cells and matched control cells *in vitro*. Comparative metabonomic profiling revealed statistically significant perturbations in key metabolites associated with amino acids and energy-associated pathway. BDV may be manipulating the host cell’s metabolic network to support viral replication and proliferation through better enabling intracellular trafficking, viral protein expression, and *de novo* lipid synthesis. However, further studies are required to validate this assertion. Through elucidating the intracellular metabolic changes incident to BDV infection *in vitro*, this study also provides direction for future exploration on the pathogenic mechanism of BDV infection *in vivo*.

## Materials and Methods

### Materials

Human OL cells (a cell line derived from fetal human oligodendrocytes), the BDV Hu-H1 strain (passages 75 and 76 in OL cells) [13] and BDV-specific nucleoprotein p40 monoclonal antibody [43] were kindly supplied by Hanns Ludwig (Free University of Berlin, Germany). The virus strain BDV Hu-H1 is one out of the first three human strains all of which are differing from the genetic profile of the laboratory reference strains V and C6BV [44]. Dulbecco’s Modified Eagle’s Medium (DMEM), fetal bovine serum (FBS), penicillin-streptomycin solution (10.000 unit/ml penicillin, 10.000 ug/ml streptomycin), L-glutamine, Phosphate Buffer Saline (PBS) (1×) and 0.25% trypsin-EDTA (1×) were purchased from Hyclone. Ultrapure water was prepared through the Millipore MilliQ purification system. Analytical grade methanol, chloroform and methanal were purchased from the Chongqing Chuandong Chemical (Group) Co., Ltd. Previous basic descriptions on how to grow, titrate, and immunostain BDV infected cells and to prepare virus stocks are adapted as described below.

### OL Cell Cultivation

A human OL cell line infected with BDV Hu-H1 strain was grown in DMEM with 10% FBS. The cells were cultured in 10-cm dishes within a humidified incubator (5% CO_2_, 37°C), and were passaged when they reached 90% confluence by trypsinization. Briefly, the passaging method involved washing the cell monolayer twice with 5.0 ml of PBS (1×) followed by a 2.0 ml trypsin rinse. Thereafter, the cells were incubated at 37°C for 1 min to allow cell detachment. Then, an equal volume of fresh growth medium was added to suspend the detached cells in solution as well as to stop trypsin action. The resultant suspension was split into portions for further subculturing.

### BDV Hu Strain Solution Preparation

The cells were sub-cultured into 20 separate 10-cm dishes. When all dishes reached 95% confluence, cells were washed twice with 5.0 ml serum-free DMEM followed by trypsin digestion for 1 min at 37°C. The trypsin was decanted, and 2.0 ml serum-free DMEM was added to each dish to suspend the detached cells in solution. Then, the cell solution was frozen (−80°C) and thawed (25°C) for three consecutive cycles, transferred to 15 ml centrifuge tubes and centrifuged at 3000 rpm for 10 min. The supernatant (containing infectious viral particles) was used as the stock viral solution, and the debris at the bottom of the tube (containing cell matter) was discarded.

### BDV Titration

A 96-well plate (Costar) was coated with 50 µl poly-lysine per well for 30 min followed by seeding with 3×10^4^ cells per well. Eight hours post-adherence, the medium was removed and 20 µl viral solution was added to each well. The viral solution was serially diluted ten-fold for 5 times, and each concentration level had four replicates. The cells were cultured for seven days in DMEM-2% FBS, during which the cell medium was replaced once every two days to maintain the extracellular environment. Viral titration was assessed by immunohistochemistry.

### BDV Infection of OL Cells

Non-infected human OL cells were subcultured in 28 separate 10-cm dishes. 14 dishes were infected with BDV (stock solution of OL cells infected with BDV Hu-H1 as above) at a multiplicity of infection (MOI) of 1.0. Specifically, the cells were washed twice with serum-free DMEM before 800 µl of stock viral solution was added into each dish. The cells were then stored in a humidified incubator (5% CO_2_, 37°C) for two hours, with gentle shaking for 15 min. Excess virus was removed by washing with 5 ml of serum-free DMEM before bathing the cells in 10 ml of culture medium (10% FBS in DMEM). The remaining 14 dishes were maintained as uninfected control OL cells. Both cell lines were then incubated under the same conditions for the remainder of the study.

### Immunoﬂuorescence Assay

Both BDV-infected and control OL cells were grown on six-well dishes for 30 min at room temperature with 4% paraformaldehyde followed by permeabilization for 5 min in 0.4% Triton X-100. Thereafter, both lines were rinsed with PBS and blocked with 5% (w/v) skimmed milk solution for one hour at 37°C. Overnight incubation with anti-BDV-specific p40 antigen primary monoclonal antibody [43] at 4°C was followed by one hour incubation with secondary antibodies at room temperature. After three PBS washes, immunofluorescence was detected by using a phase-contrast microscopy.

### Metabolite Extraction

On day 14 post-infection, cells were washed twice with 5.0 ml serum-free DMEM followed by trypsin digestion for 1 min at 37°C. The trypsin was decanted, and 2.0 ml serum-free DMEM was added to each dish to suspend the detached cells in solution. Then, the cell solution was transferred to 15 ml centrifuge tubes and centrifuged at 3000 rpm for 10 min. The supernatant was discarded, and the remaining cell pellets stored at 4°C. Analytical grade methanol and chloroform in a 2∶1 ratio (v/v; 250 µl/cell pellet) at 4°C were added to the cell pellets. The cell pellet-solvent mixture was sonicated. After approximately 15 min in contact with the first solvents, chloroform and distilled water were added to the samples in a 1∶1 ratio (250 µl/cell pellet) to form an emulsion. The samples were then centrifuged at 13000 rpm for 20 min. The supernatant was collected, lyophilized, and stored at −80°C for later ^1^H NMR spectroscopic analysis.

### ^1^H NMR Spectroscopy

The lyophilized samples were reconstituted with 600 µl D_2_O and centrifuged at 12000 rpm for 10 min at 4°C; then, 550 µl aliquots of the supernatant were pipetted into 5 mm NMR tubes for ^1^H NMR experiments. All NMR readings were performed in a Bruker AVANCE III 600 spectrometer operating at 600.13 MHz ^1^H frequency and equipped with a triple resonance probe. Standard one-dimensional (1-D) single-pulse spectra were acquired at 298 K using a 90° flip angle with a spectral width of 12000 Hz and 32 K data points. The acquisition time was 2.66 s per scan, and an additional 8 s relaxation delay (including a 2 s water signal resaturation) was used to ensure full relaxation. The total number of scans was 512. The free induction decay (FID) was zero-filled to 64 K, and an exponential line-broadening function of 0.3 Hz was applied to the FID prior to Fourier transformation. All spectra were phase and baseline corrected and referenced to the methyl peak of lactate (CH_3_, δ1.33) using the software package Topspin (v2.1 pl4, Bruker Biospin, Germany).

### Chemometric Analysis

Using Topspin, all spectra were divided into 1100 integrated regions of equal width (0.01 ppm “buckets”) corresponding to the spectral region of δ10.0 to δ-1.0. The region of δ5.1 to δ4.5 was set to a zero integral to eliminate the variation produced by both water resonance suppression and partial cross-saturation urea resonance. The remaining spectral segments of each NMR spectrum were normalized to the total integral of the spectral area on a sample-by-sample basis to partially compensate for the minor differences in metabolite concentrations across samples within each group. Chemometric analysis on the data sets was performed using the software package SIMCA-P+ (v12.0.1, Umetrics AB, Umea, Sweden).

### Statistical and Network Analysis

The main metabolites responsible for class discrimination were manually calculated by peak integration. The Wilk-Shapiro test was performed to determine normality. For parameters of normal distribution, the independent sample *t*-test was used to find significantly differentiated metabolites. For the data that did not achieve normality, the non-parametric Mann-Whitney U-test test was used. The Benjamini-Hochberg method (*q*-value) was conducted on the relative peak intensity data to control for false discovery [45]. A p-value and *q*-value ≤0.05 were considered to be statistically significant. Compounds were identified based on known chemical shifts published in online databases. The Human Metabolome Database (HMDB) [23], [46] and the BioMagResBank (BMRB) [25] were used for the NMR spectral data. The differential compounds and their respective fold changes were then inputted into the KEGG and Metacore (GeneGo) databases for network analysis.
